# Inhibition of E-Selectin (GMI-1271) or E-selectin together with CXCR4 (GMI-1359) re-sensitizes multiple myeloma to therapy

**DOI:** 10.1038/s41408-019-0227-3

**Published:** 2019-08-20

**Authors:** Barbara Muz, Feda Azab, Mark Fiala, Justin King, Daniel Kohnen, William E. Fogler, Ted Smith, John L. Magnani, Ravi Vij, Abdel Kareem Azab

**Affiliations:** 1 Department of Radiation Oncology, Cancer Biology Division, Washington University in St. Louis School of Medicine, St. Louis, MO USA; 2 Department of Medicine, Division of Medical Oncology, Washington University in St. Louis School of Medicine, Saint Louis, MO USA; 3 GlycoMimetics Inc., Rockville, MD USA

**Keywords:** Myeloma, Cancer microenvironment

Dear Editor,

Multiple myeloma (MM) is a plasma cell malignancy localized in the bone marrow (BM) characterized by continuous metastasis. MM remains incurable, despite the improved overall survival of MM patients due to clinically introduced novel treatments such as proteasome inhibitors (bortezomib and carfilzomib) and immunomodulatory drugs (IMiDs; lenalidomide)^1^.

The complex cross-talk between MM cells and the tumor microenvironment (TME) facilitates tumor progression and therapy resistance^2^. Adhesion molecules such as selectins^3,4^ and C-X-C chemokine receptor type 4 (CXCR4, CD184)^5–7^ play pivotal roles in advancement of MM, suggesting that these molecules could be targeted to reduce metastasis and overcome drug resistance. Elevated CXCR4 expression enables MM cells’ dissemination from the primary tumor site to the peripheral blood (PB) and further to a new BM site attracted by chemokines such as stromal-derived growth factor-1 (SDF-1). Homing of MM is initiated through interaction between ligands present on MM cells such as cutaneous lymphocyte-associated antigen (CLA; role of which is not well described in MM)^3,8^ and adhesion molecules E- and P-selectin expressed on vascular endothelium, which induce rolling, adhesion, and extravasation. Most of cancer therapies consist of targeting the MM cells directly; however, recently disrupting MM interactions with the TME through inhibiting adhesion of cancer cells to the TME or hindering the homing of already circulating tumor cells thus preventing metastasis have been suggested as therapeutic strategies^3–5,7,8^. In this study, we tested the effect of blocking E-selectin alone using GMI-1271^8^ and blocking both E-selectin and CXCR4 using GMI-1359^9^ on TME disruption in order to sensitize MM cells to chemotherapy.

First, analysis of *CXCR4*, *CLA*, and *E-selectin* mRNA expression in CD138+ plasma cells isolated from newly diagnosed MM patients using the Gene Expression Omnibus database^10^ showed (mean ± s.e.m.) 18,223.19 ± 452.04, 3472.67 ± 118.01, and 42.16 ± 2.01, respectively **(**Fig. 1a**)**. Interestingly, *CLA* expression in CD138+ plasma cells was the highest among patients with MM (3472.67 ± 118.01) compared to monoclonal gammopathy of undetermined significance (2459.96 ± 270.13) and healthy donors (1910.85 ± 107.72) (Fig. 1b). This data suggests that *CXCR4* mRNA is highly expressed in newly diagnosed MM patients and that *CLA* mRNA expression significantly increases with MM advancement. We have previously demonstrated that MM progression and dissemination was hypoxia dependent, and hypoxic MM cells had improved cell trafficking^6^, increased tumor initiation, and induced drug resistance contributing to minimal residual disease and relapse^7,11^. Natoni et al. demonstrated that the CLA was increased in hypoxic MM cells implying disease progression, and CLA expression was further augmented in MM cells from relapsed/refractory patients compared to newly diagnosed patients^8^. These results suggest that CLA undergoes dynamic changes with MM development and could potentially be a biomarker of disease progression and drug resistance. However, we found that CLA expression was negligible and restricted to a small subpopulation of MM cells (1.3% MM.1S, 0.9% H929 and 6.3% U266) (Fig. 1c) possibly due to lack of TME and hypoxic conditions. In all, 85% of MM.1S, 44% of H929, and 97% of U266 cells were positive for CXCR4 (Fig. 1c), with the relative mean fluorescent intensity (RMFI) of 5.8, 10.6, and 12.9, respectively (Fig. 1d). Regarding MM-supporting cells, 96% of human umbilical vein endothelial cells (HUVECs), 70% of MSP-1, and 80% of HS-5 cells were E-selectin positive (Fig. 1e) with an RMFI of 25, 12, and 17, respectively (Fig. 1f). These cells had insignificant levels of CLA; however, CXCR4 was present in HUVECs, MSP-1, and HS-5 at 57, 55, and 29% of cells, respectively (Fig. 1e), with an RMFI of 6, 3.5, and 2.9, respectively (Fig. 1f), confirming high expression of E-selectin and CXCR4 in endothelial and stromal cells.

**Fig. 1:**
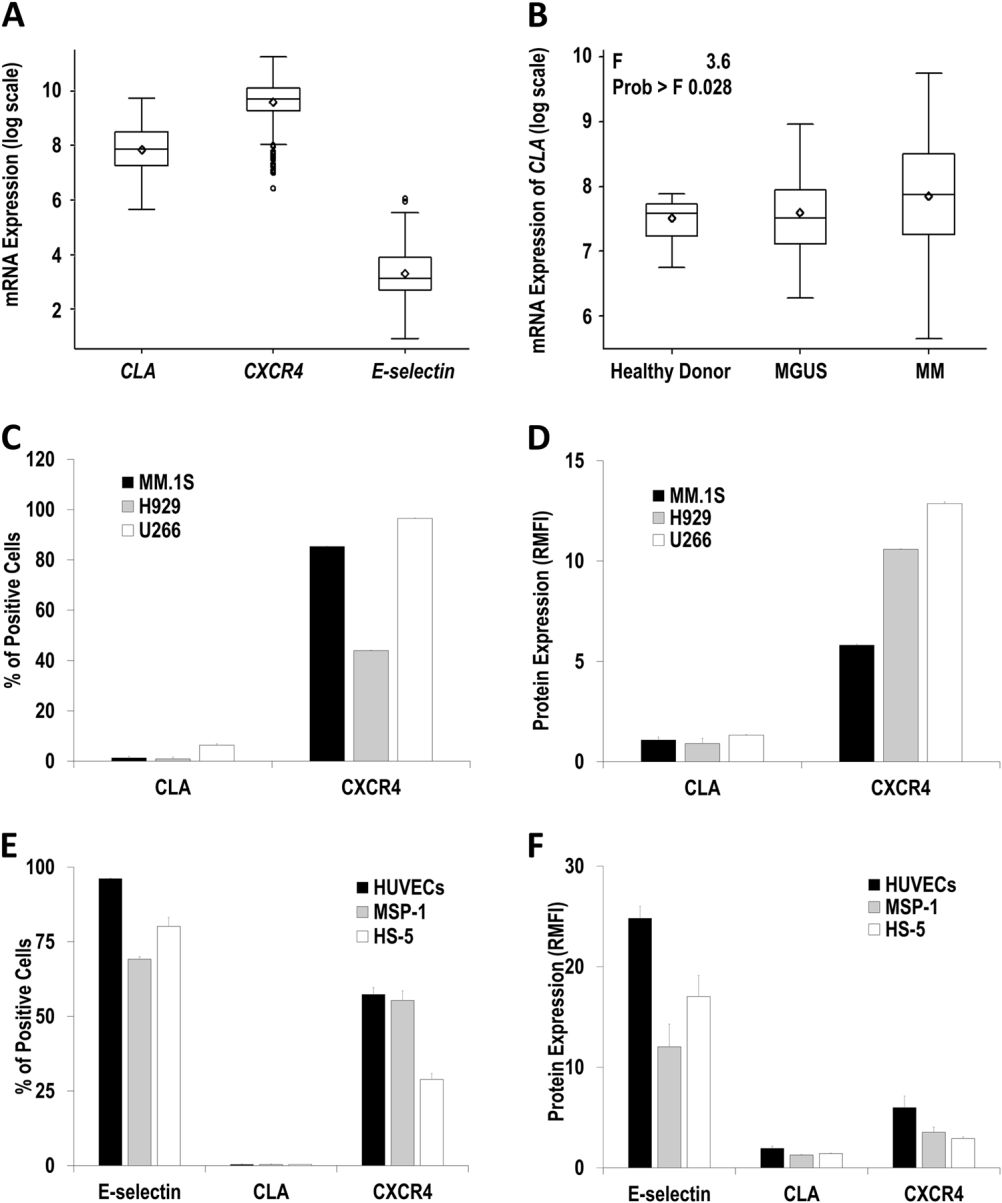
*CXCR4* mRNA is highly expressed in primary multiple myeloma (MM) cells, *CLA* expression increases with myeloma progression; CXCR4 protein expression is widely present in MM cells, while E-selectin protein is highly expressed in endothelial cells and stromal cells. Gene expression of *E-selectin* (ID 206211_at), *CLA* (ID 209879_at), and *CXCR4* (ID 217028_at) mRNA analyzed in CD138+ bone marrow plasma cells isolated from newly diagnosed MM patients (*n* = 559) using the Gene Expression Omnibus database [log scale] (**a**). The expression of *CLA* mRNA in CD138+ plasma cells harvested from healthy donors (*n* = 22), MGUS patients (*n* = 44), and newly diagnosed MM patients (*n* = 559) using the Gene Expression Omnibus database [log scale] analyzed using one-way analysis of variance test (**b)**. Percentage of CLA+- and CXCR4+-positive cells in MM cell lines demonstrated as mean ± s.e.m (**c**). CLA and C-X-C chemokine receptor type 4 (CXCR4) protein expression levels in MM cell lines demonstrated as RMFI (mean ± s.e.m.). Results are obtained from three independent biological replicates and repeated minimum in three separate experiments (**d**). Expression of cell surface proteins including E-selectin, CLA, and CXCR4 in endothelial cells (human umbilical vein endothelial cells), malignant stromal cells (MSP-1), and normal stromal cells (HS-5) demonstrated as the percentage of positive cells (**e**) and RMFI (**f**). Results are representative of at least three independent experiments and demonstrated as mean ± s.e.m. CLA cutaneous lymphocyte-associated antigen, MGUS monoclonal gammopathy of undetermined significance, RMFI relative mean fluorescent intensity

Next, we tested the effect of GMI-1271 (E-selectin antagonist) and GMI-1359 (E-selectin and CXCR4 dual antagonist) on the ability of MM cells to migrate to conditioned media. Pretreatment of MM.1S cells with GMI-1271 significantly reduced chemotaxis to HS-5 and MSP-1 media by ≤20%, while GMI-1359 greatly reduced chemotaxis to SDF-1-enriched-, HS-5-, and MSP-1-derived media by 47, 53, and 61%, respectively (Fig. 2a). U266, but not MM.1S and H929, cell adhesion to HUVECs was significantly reduced following GMI-1271 treatment, possibly due to the highest percentage of CLA-positive cells, while GMI-1359 treatment significantly decreased adhesion of MM.1S, H929, and U266 to HUVECs by 38, 53, and 42%, respectively (Fig. 2b). Furthermore, in order to recapitulate the MM extravasation through the endothelium to the BM, we performed a trans-endothelial migration in vitro, where labeled MM cells were added on top of HUVECs and trans-migrated toward stromal cells cultured at the bottom of a Boyden chamber. MM cells cultured overnight in hypoxia, which was shown to increase CXCR4^6^ and CLA^8^ expression, decreased the hypoxia-induced trans-endothelial migration of MM cells to stroma when pretreated with GMI-1271 or GMI-1359 by 17% and 40%, respectively (Fig. 2c). Similarly, GMI-1359 pretreatment caused a significant MM.1S cell retention in the circulation and prevented extravasation at 90 min postintravenous injection in vivo (Fig. 2d). Since GMI-1359 blocks not only CXCR4 but also E-selectin, we performed an experiment where either MM cells or mouse endothelium or both were pretreated with GMI-1359 before monitoring MM homing to the BM (Fig. 2e). When mice were injected with GMI-1359 intravenously 30 min prior to the injection of labeled MM cells, homing of the MM cells was inhibited by ~40% compared to vehicle-treated mice. Moreover, when the cells were pretreated with GMI-1359 prior injection, their homing to the BM was almost completely abrogated, regardless whether the mice endothelium was pretreated with GMI-1359 or not. Interestingly, U266 homed the least compared to MM.1S and H929.

**Fig. 2:**
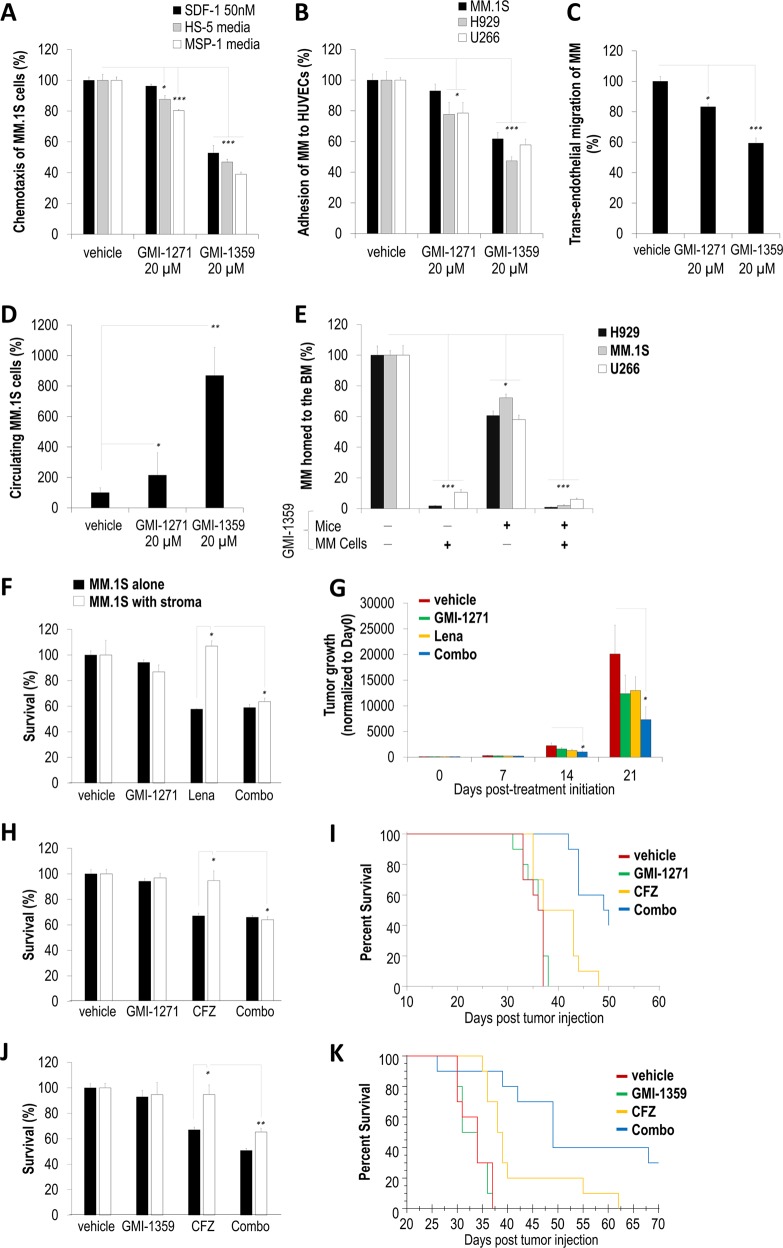
GMI-1271 and GMI-1359 disrupt the TME–multiple myeloma (MM) cell interaction by decreasing C-X-C chemokine receptor type 4 (CXCR4) and E-selectin-mediated adhesion and chemotaxis of MM cells, which sensitizes MM cells to therapy in vitro, and by inhibiting the homing process thus extending the exposure of MM cells to chemotherapies, delaying tumor growth, and improving mice survival in vivo. Percentile of vehicle-, GMI-1271-, or GMI-1359-treated MM.1S migrated toward conditioned media derived from either MSP-1 or HS-5 or 10% fetal bovine serum/RPMI1640 media enriched with stromal-derived growth factor-1 (50 nM) and normalized to vehicle-treated MM.1S (**a**). Percentile of vehicle-, GMI-1271-, or GMI-1359-treated MM.1S, H929, and U266, which adhered to human umbilical vein endothelial cells (HUVECs), normalized to vehicle-treated MM cells (**b**). Percentile of vehicle-, GMI-1271-, or GMI-1359-treated MM.1S trans-migrated through HUVECs to MSP-1 stromal cells and normalized to vehicle-treated MM cells (**c**). Percentile of vehicle-, GMI-1271-, or GMI-1359-treated MM.1S cells, which remained in the circulation of mice at 90 min post-MM injection (**d**). Percentile of vehicle- or GMI-1359-pretreated H929, MM.1S, and U266 cells, which homed to the BM in vehicle- or GMI-1359-pretreated mice (i.e., injected intravenously with 40 mg/kg of GMI-1359 30 min before MM injection) counted in the BM by flow cytometry demonstrated as mean ± s.e.m. (**e**). Survival of MM.1S cells treated with or without GMI-1271 (20 µM) and lenalidomide (1 µM) for 24 h cultured with or without stromal cells (recapitulating TME) tested by 3-[4,5-dimethylthiazol-2-yl]-2,5 diphenyl tetrazolium bromide (MTT) assay (**f**). Tumor progression of MM.1S-GFP-Luc cells injected into SCID mice treated with: (1) vehicle, (2) GMI-1271 (40 mg/kg), (3) lenalidomide (25 mg/kg), and (4) a combination of lenalidomide (25 mg/kg) and GMI-1271 (40 mg/kg) followed weekly using bioluminescence imaging normalized to Day 0 posttreatment initiation (**g**). Survival of MM.1S cells treated with or without GMI-1271 (20 µM) and carfilzomib (CFZ; 5 nM) for 24 h cultured with or without stromal cells (recapitulating TME) tested by MTT assay (**h**). Percentile of MM-bearing mice survival receiving: (1) vehicle, (2) GMI-1271 (40 mg/kg), (3) CFZ (3 mg/kg), and (4) a combination of GMI-1271 (40 mg/kg) and CFZ (3 mg/kg) followed daily, and demonstrated as a Kaplan–Meier curve. The survival of mice receiving CFZ as a single drug or GMI-1271 in combination with CFZ was significantly extended compared to vehicle (*p* = 0.0274 and *p* = 0.0001, respectively); additionally, GMI-1271 in combination with CFZ significantly extended mice survival compared to CFZ only (*p* = 0.0006) (**i**). Survival of MM.1S cells treated with or without GMI-1359 (20 µM) and CFZ (5 nM) for 24 h cultured with or without stromal cells (recapitulating TME) tested by MTT assay (**j**). Percentile of MM-bearing mice survival receiving: (1) vehicle, (2) GMI-1359 (40 mg/kg), (3) CFZ (3 mg/kg), and (4) a combination of GMI-1359 (40 mg/kg) and CFZ (3 mg/kg) followed daily, and demonstrated as a Kaplan–Meier curve. The survival of mice receiving CFZ as a single drug or GMI-1359 in combination with CFZ was significantly extended compared to vehicle (*p* = 0.0002 and *p* = 0.0001, respectively). In addition, GMI-1359 in combination with CFZ significantly extended mice survival compared to CFZ only (*p* = 0.014) (**k**). In vitro studies were performed in quadruplets, repeated minimum three times, and demonstrated as mean ± s.e.m. with statistical significance for *p* values <0.05 calculated using unpaired Student’s *t* test (*p < 0.05; **p < 0.01; ***p < 0.001). Statistical analysis for in vivo experiments was performed using two-way analysis of variance. BM bone marrow, TME tumor microenvironment

Next, we examined the effect of GMI-1271 in combination with lenalidomide on MM.1S survival cultured with or without MSP-1 stromal cells in vitro. We found that GMI-1271 alone did not affect MM.1S survival, and co-culture with stroma significantly induced drug resistance to lenalidomide, while combination treatment with both drugs significantly overcame the stroma-induced lenalidomide resistance (Fig. 2f). Similar results were observed for H929 survival co-cultured with HUVECs (Supplementary Fig. 1A). Consequently, we tested MM tumor progression in a human xenograft mouse model, where SCID mice were inoculated with MM.1S-Luc and tumor progression was monitored using bioluminescent imaging. GMI-1271 and lenalidomide as single agents delayed tumor growth by 40% and 35%, respectively, while combined lenalidomide and GMI-1271 significantly delayed tumor growth by 55% and 64% at days 14 and 21, respectively, compared to vehicle (Fig. 2g). Next, we tested GMI-1271 in combination with carfilzomib (CFZ) on MM.1S survival in vitro, which significantly overcame the stroma-induced CFZ resistance (Fig. 2h). Similar results were obtained for H929 and U266 co-cultured with stroma, treated with GMI-1271 in combination with CFZ and bortezomib (BTZ) (Supplementary Fig. 1). Subsequently, we examined mice survival using a syngeneic 5TGM1 disseminated mouse model and demonstrated significantly extended median survival in groups treated with vehicle, GMI-1271, CFZ, or combination, which were 36.5, 36.5, 40, and 49.5 days, respectively (Fig. 2i). Next, combination treatment with GMI-1359 and CFZ studied in vitro demonstrated that GMI-1359 significantly overcame the stroma-induced resistance to CFZ (Fig. 2j). Similar results were obtained for H929 and U266 co-cultured with stroma and treated with GMI-1359 in combination with CFZ or BTZ (Supplementary Fig. 2), as well as using a three-dimensional tissue-engineered bone marrow with MM.1S co-cultured with accessory cells recapitulating TME (Supplementary Fig. 3). Subsequently, median survival of mice inoculated with 5TGM1 cells treated with vehicle, GMI-1359, CFZ, or combination was significantly extended to 32.5, 34, 38.5, and 49 days, respectively (Fig. 2k). These results imply that E-selectin and/or CXCR4 antagonists (GMI-1271 and GMI-1359) were sufficient in retaining MM cells in the circulation, inferring longer MM exposure to chemotherapies and improved MM response to proteasome inhibitors and IMiDs. Our results are in agreement with others showing that both GMI-1271 and GMI-1359 disrupted the TME and mobilized cancer cells into the circulation more effectively and gradually over long periods of time compared to CXCR4 inhibition alone (using Plerixafor)^8,12–14^. Again, sustaining the presence of tumor cells in the blood by inhibiting their re-entry into the BM provides a longer window to target these cells in the circulation.

In conclusion, majority of MM patients relapse and become refractory to therapy, due to the supportive TME that assists cancer with drug resistance and metastasis^1,2^. Herein, we disrupted the interaction between MM cells and the TME with GMI-1271 and GMI-1359 by decreasing CXCR4 and E-selectin-mediated adhesion and chemotaxis of MM cells, which sensitized them to therapy in vitro, and by inhibiting the homing process thus extending the exposure of MM cells to chemotherapies and improving mice survival in vivo. These results provide preclinical basis for future clinical trials to test the ability of dual inhibition of E-selectin and CXCR4 to sensitize relapsed/refractory MM patients to therapy. Notably, a completed Phase 1/2 clinical trial in acute myeloid leukemia patients using GMI-1271 showed high remission rates and improved overall survival with favorable safety^15^, and a Phase 1 clinical trial is being performed on MM patients (NCT02811822). Currently, GMI-1359 is being evaluated in a Phase 1 clinical trial in healthy volunteers (NCT02931214).

## Supplementary information

Supplemental

Supplementary figures.

## References

[1] Kumar SK (2017). Multiple myeloma. Nat. Rev. Dis. Primers.

[2] de la Puente P (2015). 3D tissue-engineered bone marrow as a novel model to study pathophysiology and drug resistance in multiple myeloma. Biomaterials.

[3] Azab AK (2012). P-selectin glycoprotein ligand regulates the interaction of multiple myeloma cells with the bone marrow microenvironment. Blood.

[4] Muz B (2015). Inhibition of P-Selectin and PSGL-1 using humanized monoclonal antibodies increases the sensitivity of multiple myeloma cells to bortezomib. Biomed Res. Int..

[5] Azab AK (2009). CXCR4 inhibitor AMD3100 disrupts the interaction of multiple myeloma cells with the bone marrow microenvironment and enhances their sensitivity to therapy. Blood.

[6] Azab AK (2012). Hypoxia promotes dissemination of multiple myeloma through acquisition of epithelial to mesenchymal transition-like features. Blood.

[7] Muz B, de la Puente P, Azab F, Luderer M, Azab AK (2014). Hypoxia promotes stem cell-like phenotype in multiple myeloma cells. Blood Cancer J..

[8] Natoni A (2017). E-selectin ligands recognised by HECA452 induce drug resistance in myeloma, which is overcome by the E-selectin antagonist, GMI-1271. Leukemia.

[9] Zhang W (2016). Dual E-Selectin/CXCR4 antagonist GMI-1359 exerts efficient anti-leukemia effects in a FLT3 ITD mutated acute myeloid leukemia patient-derived xenograft murine model. Blood.

[10] Zhan F (2006). The molecular classification of multiple myeloma. Blood.

[11] Muz B (2016). A CD138-independent strategy to detect minimal residual disease and circulating tumour cells in multiple myeloma. Br. J. Haematol..

[12] Fogler WE (2016). Administration of the dual E-Selectin/CXCR4 antagonist, GMI-1359, results in a unique profile of tumor mobilization from the bone marrow and facilitation of chemotherapy in a murine model of FLT3 ITD AML. Blood.

[13] Natoni A (2014). Multiple myeloma cells express functional E-Selectin ligands which can be inhibited both in vitro and in vivo leading to prolongation of survival in a murine transplant model. Blood.

[14] Glavey SV (2014). The sialyltransferase ST3GAL6 influences homing and survival in multiple myeloma. Blood.

[15] DeAngelo, D. J. et al. GMI-1271 improves efficacy and safety of chemotherapy in R/R and newly diagnosed older patients with AML: results of a phase 1/2 study. *Blood* **130**, 894 (2017).

