# Supplementary material for: Inhibition of E-Selectin (GMI-1271) or E-selectin together with CXCR4 (GMI-1359) re-sensitizes multiple myeloma to therapy
Source: Blood Cancer J. 2019 Aug 20;9(9):68. doi: 10.1038/s41408-019-0227-3 (PMC6702213; doi:10.1038/s41408-019-0227-3)
Supplement: Supplementary file 1 — Supplemental [file 41408_2019_227_MOESM1_ESM.docx]

**MATERIALS AND METHODS:**

**Gene expression analysis**

Gene expression of *CXCR4* (probe ID 217028_at), Cutaneous Lymphocyte Antigen, *CLA* (probe ID 209879_at) and *E-selectin* (probe ID 206211_at) in CD138+ plasma cells isolated from newly diagnosed MM patients (n=559), was analyzed using publicly available the Gene Expression Omnibus database (Zhan et al, Blood 2006). The expression of *CLA* in CD138+ plasma cells from healthy donors (n=22), patients with Monoclonal Gammopathy of Undetermined Significance, MGUS (n=44), and newly diagnosed MM patients (n=559) was analyzed using the same database, and demonstrated in the log scale.

**Cell culture**

MM cell lines (MM.1S, H929, U266, MM.1S-GFP-Luc and 5TGM1; ATCC, Manassas, VA) were cultured in RPMI-1640 medium (Corning CellGro, Mediatech, Manassas, VA), supplemented with 10% fetal bovine serum (FBS, Gibco, Life Technologies, Grand Island, NY), 2 mmol/l L-glutamine, 100 U/ml Penicillin and 100 μg/ml Streptomycin (CellGro, Mediatech, Manassas, VA). Human umbilical vein endothelial cells (HUVECs) were purchased from (AngioProteomie, Atlanta, GA) and were cultured in endothelial cell growth media containing supplements (AngioProteomie). Human normal stromal cells (HS-5, purchased from ATCC) and the malignant MSP-1 stromal cell line derived from MM patients’ BM biopsy (de la Puente et al, Haematologica 2016) were cultured in Dulbecco’s Modified Eagle’s Medium (DMEM; Corning CellGro) containing 10% FBS, L-glutamine, Penicillin and Streptomycin (as above). Cells were cultured at 37°C and 5% CO_2_ in a humidified tissue culture incubator (21% O_2_, NuAire water jacket incubator, Plymouth, MN) or hypoxic conditions (1% O_2_, Coy, Grass Lake, MI). All cell lines were authenticated using respective markers and tested for mycoplasma using PlasmoTest Mycoplasma Detection Kit (InvivoGen; San Diego, CA).

**E-selectin, CLA and CXCR4 protein expression**

MM cell lines, HS-5, MSP-1, and HUVECs were stained with anti-human CD62E/E-selectin-PE (Cat# 551145; BD Biosciences, San Jose, CA), anti-human CXCR4-APC (Cat# 555976) or anti-human CLA (HECA452 clone; a monoclonal antibody that recognises SLex-related structures that bind to E-selectin)-FITC (Cat# 555947) antibodies with respective isotype controls (all antibodies purchased from BD Biosciences) for 1 hr on ice, and the expression was analyzed with MACSQuant Flow Cytometer (Miltenyi, San Diego, CA). The percentage of CLA or CXCR4 positive cells were detected comparing to isotype control. Expression of CLA and CXCR4 in these subpopulations were demonstrated as relative mean fluorescent intensity (RMFI) calculated by MFI of target protein divided by MFI of respective isotype control.

**Chemotaxis assay**

Chemotaxis assay and trans-endothelial migration assay were performed using an 8.0 µM pore size Boyden Chamber (HTS Transwell-96 well plates purchased from Corning, Cat# 3384). For chemotaxis, media collected from MSP-1 and HS-5 cell cultures, or media enriched with 50nM SDF-1 were added to the lower compartment (235 µl /well) of a Boyden chamber. MM.1S cells were serum-starved for 3 hr, pre-labeled with Calcein-AM, pre-treated with GMI-1271 or GMI-1359 (20 µM for 1 hr), added to the upper compartment (1 × 10^5^ cells /75 µl /well), and allowed to migrate towards conditioned media for 6 hr. Calcein-AM+ MM cells which migrated into the lower compartment were counted by flow cytometry.

Before initiating the trans-endothelial migration assay, HUVECs were cultured overnight in the upper compartment of a Boyden chamber (5 × 10^3^ cells /well), and MSP-1 in the lower compartment (5 × 10^3^ cells/ 235µl/ well). MM.1S cells were incubated in hypoxia (1% O_2_) for 24 hr, serum-starved for 3 hr, pre-labeled with Calcein-AM, treated with GMI-1271 or GMI-1359 (20 µM for 1 hr), then added to the upper compartment (1 × 10^5^ cells /75µl /well), and allowed to trans-migrate for 6 hr. Calcein-AM+ MM cells which migrated through endothelium towards the MSP-1 cells in the lower compartment were counted by flow cytometry.

**Adhesion assay**

HUVECs (5 × 10^3^ cells/well) were cultured overnight to confluence in 96-well black plates before initiating the adhesion assay. MM.1S, H929 and U266 were serum-starved for 3 hr, pre-labeled with Calcein-AM, pre-treated with GMI-1271 or GMI-1359 (20 µM for 1 hr), added to adherent cells at a concentration 1 × 10^5^ cells/ 100 µl /well), and allowed to adhere for 2 hr at 37°C. Non-adherent cells were aspirated, HUVECs were washed with 1 x PBS, and fluorescence intensity of MM adherent cells was measured using a fluorescent-plate reader (Ex/Em = 485/520 nm).

**Cell survival assay**

MM cells with or without accessory cells (HUVECs, HS-5 or MSP-1) recapitulating TME were treated with or without GMI-1271 (20 µM) or GMI-1359 (20 µM), alone or in combination with lenalidomide (1 µM), carfilzomib (CFZ; 5nM) and bortezomib (BTZ, 5nM) (Selleck Chem, Houston, TX). Cells survival was analyzed by MTT assay according to manufacturers’ protocol. Briefly, after 24 hr of treatment, 10 µL of MTT solution [3-(4,5-dimethylthiazol-2-yl)-2,5-diphenyltetrazolium bromide] (Sigma-Aldrich, St. Louis, MO), was added to the cells for 3 hr, then the stop solution was added and the formazan crystals were solubilized overnight at 37˚C, followed by the absorbance readout at 570nm using a spectrophotometer. Survival of MM.1S-GFP cells (with or without TME) was also tested using a 3D-tissue engineered BM (3DTEBM) derived from MM patients’ BM aspirates, which included accessory cells such as immune cells, stromal cells and endothelial cells (de la Puente et al, Biomaterials, 2015). After 48 hr of treatment, the scaffolds were digested and the number of MM.1S-GFP cells was analyzed by flow cytometry.

**Cell retention and homing study *in vivo***

All animal experiments were performed in compliance with the Guidelines for Care and Use of Research Animals established by the Division of Comparative Medicine and the Animal Studies Committee of Washington University School of Medicine. MM.1S cells were pre-labeled with Calcein-Violet, Calcein-Red/Orange and Calcein-AM and pre-treated with vehicle (saline), GMI-1271 (20 µM) or GMI-1359 (20 µM) for 1 hr, respectively. Anesthetized BALB/cJ mice (n=5, female, 8 weeks old; The Jackson Laboratory, Bar Harbor, ME) were injected intravenously (IV) with the mixture of MM cells (in total 3 × 10^6^ cells/ mice of vehicle-, GMI-1271- and GMI-1359-treated in ratio 1:1:1), and 90 min post MM injection, the mice were bled from the submandibular vein and the number of circulating Calcein-Violet+, Calcein-Red/Orange+ and Calcein-AM+ MM cells was analyzed by flow cytometry. Additionally, BALB/cJ mice (n=12, female, 8 weeks old; The Jackson Laboratory) were divided into 4 groups (n=3 mice/group and 2 femurs per mouse): (i) vehicle-treated mice injected with untreated MM cell lines (H929-Calcein-AM+, MM.1S-Calcein-Violet+, and U266-Calcein-Red/Orange+; 3 x 10^6^ cells in total in the ratio of 1:1:1); (ii) vehicle-treated mice injected with GMI-1359-treated MM cells (20 µM for 1 hr); (iii) GMI-1359-treated mice (40 mg/kg, IV, 30 min prior to the injection of MM cells) injected with vehicle-treated MM cells; and (iv) GMI-1359-treated mice injected with GMI-1359-treated MM cells (as above). At 60 min after MM injection mice were sacrificed, BM extracted, and the number of homed MM cells was counted by flow cytometry.

**Tumor growth using bioluminescence imaging**

The effect of the GMI-1271 in combination with lenalidomide was tested in a disseminated xenograft mouse model. MM.1S-GFP-Luc cells (2 x 10^6^ cells/mouse) were injected IV into 40 SCID mice (strain NOD.CB17-Prkdcscid/J; female, 9 weeks old; The Jackson Laboratory). At week 4, mice were randomly allocated into 4 groups (n=10 mice per group): (1) vehicle (saline), (2) lenalidomide (25 mg/kg administered per os, PO, 5 days a week for 3 weeks), (3) GMI-1271 (40 mg/kg administered IP 5 days a week for 3 weeks), and (4) a combination of lenalidomide (25 mg/kg administered PO, 5 days a week for 3 weeks) and GMI-1271 (40 mg/kg administered IP 5 days a week for 3 weeks). Tumor progression was monitored by bioluminescence imaging once a week. Briefly, mice were first injected IP with D-Luciferin (150 mg/kg; Gold Biotechnology, St. Louis, MO), anesthetized with 2% isofluorane vaporized in oxygen and imaged 10 min post-injection with IVIS 50 (PerkinElmer, Waltham, MA). Total photon flux per sec was measured from fixed regions of interest (ROIs) drawn around the whole mouse body (excluding tail) using Living Image 2.6. The results are demonstrated as a signal intensity of ROI and normalized to day 0 post-treatment initiation.

**Mice survival study**

A syngeneic disseminated mouse model was used to study the effect of GMI-1271 or GMI-1359 in combination with carfilzomib (CFZ). Mouse MM cells, 5TGM1 (5 x 10^6^ cells/mouse), were injected into n=40 C57BL/KaLwRijHsd mice (female; 9 weeks old; Envigo RMS, Indianapolis, IN) and monitored by bioluminescence imaging. At day 16, mice were randomly allocated into 4 groups (n=10 mice per group): (1) vehicle, (2) GMI-1271 (40 mg/kg administered IP daily for 14 days; QDx14), (3) CFZ (3 mg/kg administered IV QDx2), and (4) a combination of GMI-1271 and CFZ. Similarly as described above, GMI-1359 in combination with CFZ was tested on mice survival. The efficacy of the treatment on mice survival was monitored daily by an investigator blinded to groups and demonstrated as the Kaplan–Meier plots.

**Statistics**

All data are expressed as mean ± standard error of the mean (s.e.m.). *In vitro* experiments were performed in quadruples and replicated independently at least three times, and statistical significance was analyzed using Student’s *t*-test. The analysis of *CLA* mRNA expression and *in vivo* data was analyzed using one-way ANOVA test. Values were considered significantly different for *p* values less than 0.05 (**p*<0.05; ***p*<0.01; ****p*<0.001).

1. Zhan F, Huang Y, Colla S, Stewart JP, Hanamura I, Gupta S*, et al.* The molecular classification of multiple myeloma. Blood **2006**;108(6):2020-8 doi 10.1182/blood-2005-11-013458.

2. de la Puente P, Quan N, Hoo RS, Muz B, Gilson RC, Luderer M*, et al.* Newly established myeloma-derived stromal cell line MSP-1 supports multiple myeloma proliferation, migration, and adhesion and induces drug resistance more than normal-derived stroma. Haematologica **2016**;101(7):e307-11 doi 10.3324/haematol.2016.142190.

3. de la Puente P, Muz B, Gilson RC, Azab F, Luderer M, King J*, et al.* 3D tissue-engineered bone marrow as a novel model to study pathophysiology and drug resistance in multiple myeloma. Biomaterials **2015**;73:70-84 doi 10.1016/j.biomaterials.2015.09.017.
