# Supplementary material for: Inhibition of E-Selectin (GMI-1271) or E-selectin together with CXCR4 (GMI-1359) re-sensitizes multiple myeloma to therapy
Source: Blood Cancer J. 2019 Aug 20;9(9):68. doi: 10.1038/s41408-019-0227-3 (PMC6702213; doi:10.1038/s41408-019-0227-3)
Supplement: Supplementary file 2 — Supplementary figures. [file 41408_2019_227_MOESM2_ESM.pdf]

Supplementary Figure 1

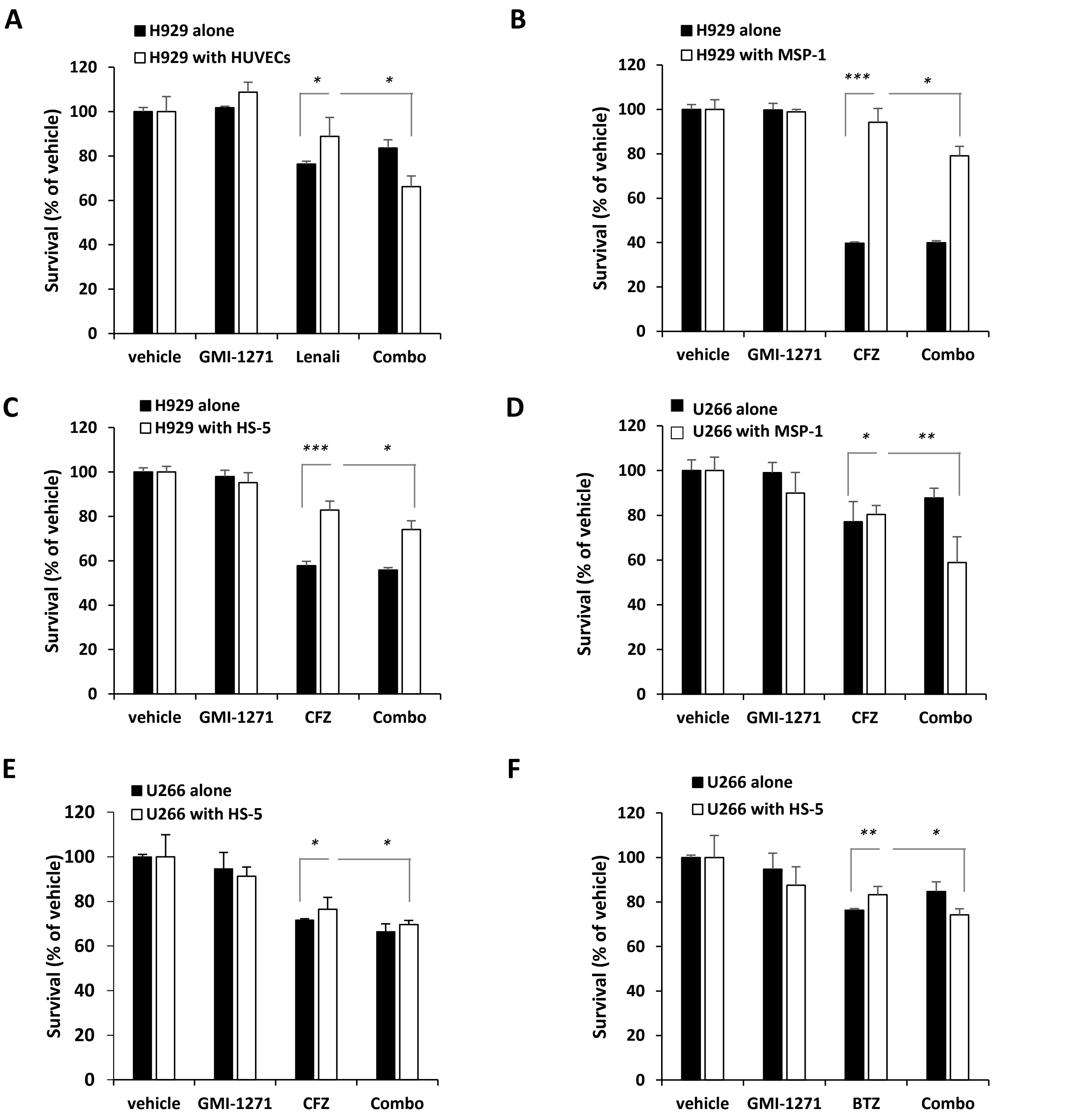

Supplementary Figure 1 Combination of GMI-1271 with lenalidomide, carfilzomib or bortezomib overcomes stroma-induced drug resistance in vitro. Survival of H929 cells treated with GMI-1271 (20 μM) and cultured with or without HUVECs +/- lenalidomide (Lenali, 1 μM) **(A)**, with or without MSP-1 +/- carfilzomib (CFZ, 5nM) **(B)**, and with or without HS-5 +/- CFZ **(C)**. Survival of U266 cells treated with GMI-1271 (20 μM) and cultured with or without MSP-1 +/- CFZ (5 nM) **(D)**, with or without HS-5 +/- CFZ **(E)**, and with or without HS-5 +/- BTZ (5 nM) **(F)**. Survival of MM cells was tested at 24 hours using MTT assay. Results are representative of at least three independent experiments and demonstrated as mean ± s.e.m. with statistical significance for p values less than 0.05 calculated using unpaired Student's t-test (*p<0.05; **p<0.01; ***p<0.001).

Supplementary Figure 2

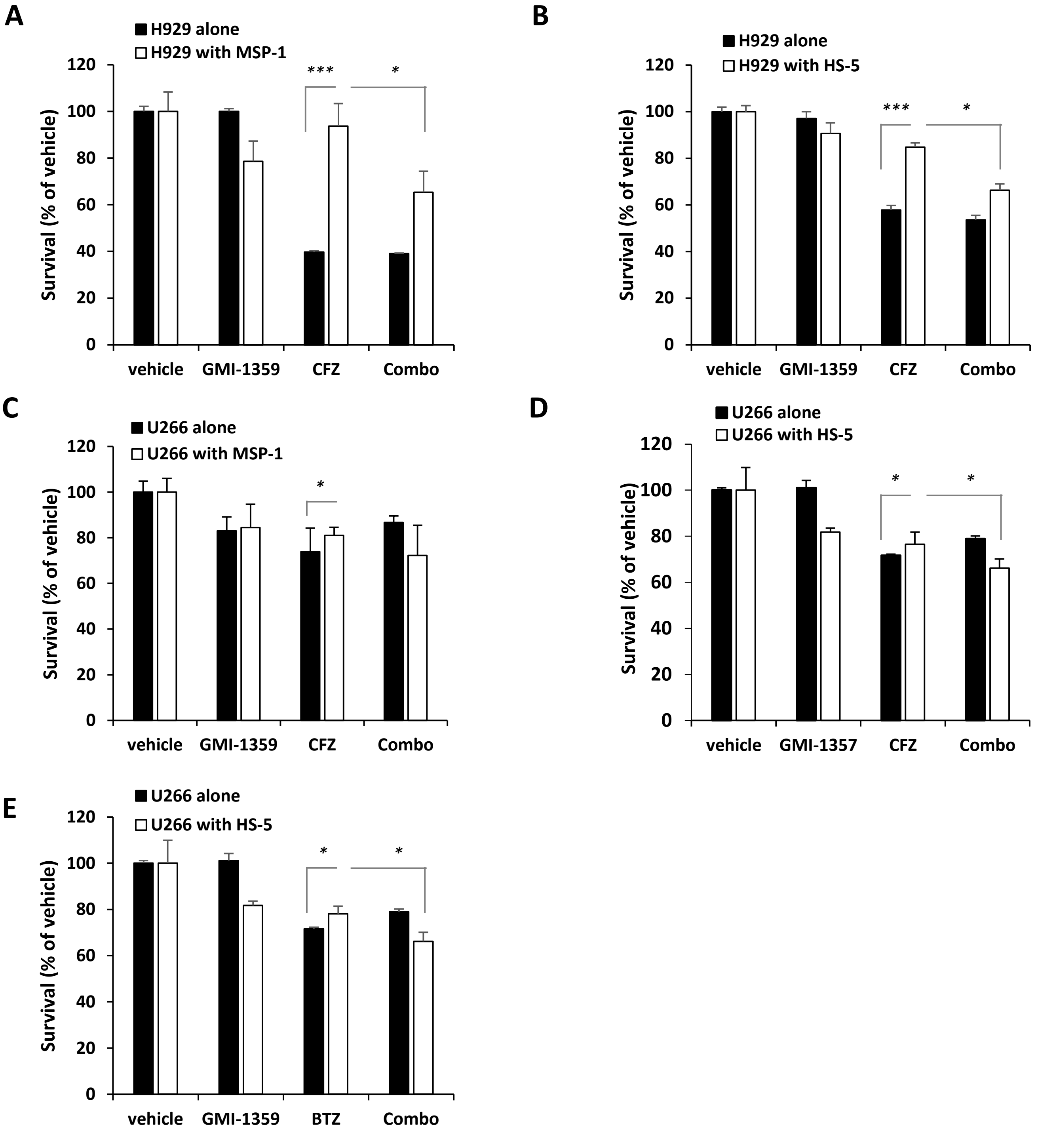

Supplementary Figure 2 Combination of GMI-1359 with carfilzomib or bortezomib overcomes stroma-induced drug resistance in vitro. Survival of H929 cells treated with GMI-1359 (20 µM) and cultured with or without MSP-1 +/- carfilzomib (CFZ, 5nM) **(A)**, and with or without HS-5 +/- CFZ **(B)**. Survival of U266 cells treated with GMI-1359 (20 µM) and cultured with or without MSP-1 +/- CFZ (5 nM) **(C)**, with or without HS-5 +/- CFZ **(D)**, and with or without HS-5 +/- BTZ (5 nM) **(E)**. Survival of MM cells was tested at 24 hours using MTT assay. Results are representative of at least three independent experiments and demonstrated as mean ± s.e.m. with statistical significance for *p* values less than 0.05 calculated using unpaired Student's *t*-test (*p<0.05; ***p<0.001).

Supplementary Figure 3

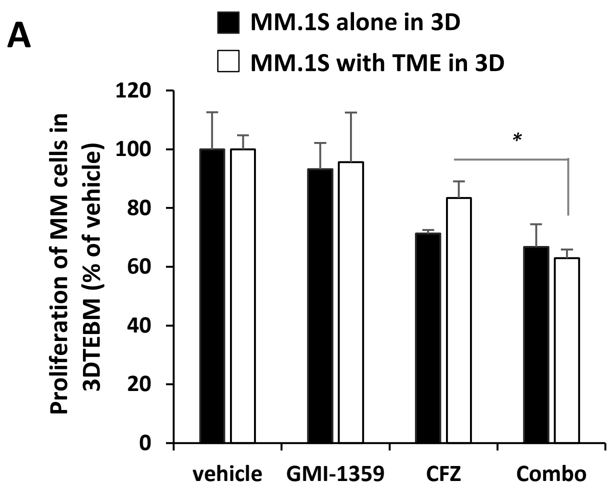

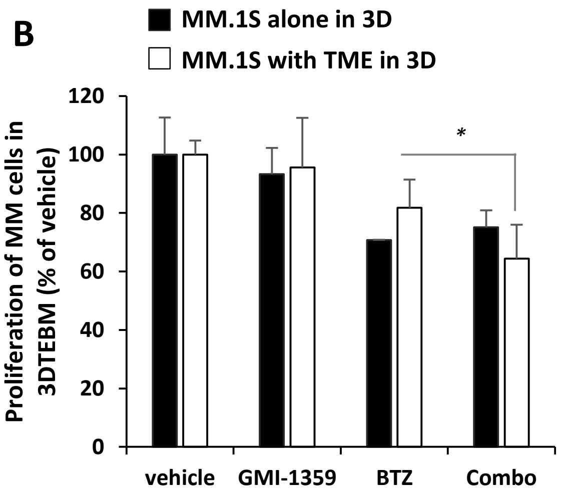

Supplementary Figure 3 Combination of GMI-1359 with carfilzomib or bortezomib overcomes stroma-induced drug resistance in vitro in the 3D tissue culture model. MM.1S-GFP cells were cultured with and without accessory cells (recapitulating tumor microenvironment, TME) and treated with or without GMI-1359 (25 µM), alone or in combination with carfilzomib (CFZ; 10 nM) **(A)** or bortezomib (BTZ; 10 nM) **(B)** in 3DTEBM for 48 hours. Scaffolds were then digested with collagenase and the number of surviving MM.1S-GFP+ cells was analyzed by flow cytometry. Results are representative of at least three independent experiments and demonstrated as mean ± s.e.m. with statistical significance for *p* values less than 0.05 calculated using unpaired Student's *t*-test (*p<0.05).
